# Loss of *Arid1a* Promotes Neuronal Survival Following Optic Nerve Injury

**DOI:** 10.3389/fncel.2020.00131

**Published:** 2020-05-15

**Authors:** Xue-Qi Peng, Shang-Kun Dai, Chang-Ping Li, Pei-Pei Liu, Zhi-Meng Wang, Hong-Zhen Du, Zhao-Qian Teng, Shu-Guang Yang, Chang-Mei Liu

**Affiliations:** ^1^State Key Laboratory of Stem Cell and Reproductive Biology, Institute of Zoology, Chinese Academy of Sciences (CAS), Beijing, China; ^2^Savaid Medical School, University of Chinese Academy of Sciences, Beijing, China; ^3^Institute for Stem Cell and Regeneration, Chinese Academy of Sciences, Beijing, China; ^4^Department of Orthopedic Surgery, The Johns Hopkins University School of Medicine, Baltimore, MD, United States

**Keywords:** *Arid1a*, SWI/SNF, retinal ganglion cell, optic nerve crush, survival

## Abstract

Trauma or neurodegenerative diseases trigger the retrograde death of retinal ganglion cells (RGCs), causing an irreversible functional loss. AT-rich interaction domain 1A (ARID1A), a subunit of the SWItch/Sucrose Non-Fermentable (SWI/SNF) chromatin remodeling complex, has been shown to play crucial roles in cell homeostasis and tissue regeneration. However, its function in adult RGC regeneration remains elusive. Here, we show that optic nerve injury induces dynamic changes of *Arid1a* expression. Importantly, deleting *Arid1a* in mice dramatically promotes RGC survival, but insignificantly impacts axon regeneration after optic nerve injury. Next, joint profiling of transcripts and accessible chromatin in mature RGCs reveals that *Arid1a* regulates several genes involved in apoptosis and JAK/STAT signaling pathway. Thus, our findings suggest modulation of *Arid1a* as a potential therapeutic strategy to promote RGC neuroprotection after damage.

## Introduction

Optic nerve trauma (e.g., various cranial and orbital injuries) or optic neuropathies (e.g., glaucoma and ischemia) typically lead to axon damage, progressive loss of retinal ganglion cells (RGCs), and even irreversible visual function deficits (Crair and Mason, 2016; Benowitz et al., 2017). Like most other neurons in the central nervous system (CNS), mature RGCs fail to regenerate their axons after optic nerve injury, due to the extrinsic environment inhibitory elements and the diminished intrinsic regenerative capacity (He and Jin, 2016; Laha et al., 2017). Recent studies have shown that adult RGCs can regain the intrinsic ability to support axon regeneration after optic nerve injury by manipulating reprogramming factors, such as *Klf4*, *c-myc*, and *Lin28*, etc. (Moore et al., 2009; Belin et al., 2015; Wang et al., 2018). More recently, combinatorial approaches *via* modulating multiple independent pathways have been shown to promote injured axons to regenerate beyond the optic chiasm into the brain and reform functional synapses with their original targets (Lim et al., 2016; Goulart et al., 2018). For example, visual stimulation/RHEB1 overexpression led to regenerating axons back to almost all the correct targets. However, synaptic reconnections partially restored optomotor response but not rescued pupil response, depth perception, visual fear response, suggesting that insufficient numbers of regenerating axons projection to the correct targets (Lim et al., 2016). Besides, knocking out *Bax*, a pro-apoptotic member of the *Bcl-2* family, not only promoted neuronal survival after optic nerve injury but also enabled axon regeneration despite delayed initiation of the regenerative program (Yungher et al., 2017). Therefore, long-term RGC neuroprotection after optic nerve injury is crucial to slow neurodegeneration, thereby improving the therapeutic window that might trigger axon regeneration.

Rearrangements of chromatin structure mediated by SWItch/Sucrose Non-Fermentable (SWI/SNF) are critical for modulation of gene expression, involving in many cellular processes including proliferation and differentiation (Roberts and Orkin, 2004; Euskirchen et al., 2012). Loss-of-function of AT-rich interaction domain 1A (ARID1A, also known as BAF250a), one principal component of SWI/SNF, disrupts SWI/SNF targeting and nucleosome remodeling and the resulting aberrant gene expression (Chandler et al., 2013; Kadoch et al., 2013). As a tumor suppressor, *Arid1a* is associated with the development, survival, and progression of cancer cells, and its dysfunction may be key tumorigenic events in ovarian, endometrial, gastric, and breast cancers (Zang et al., 2012; Takeda et al., 2016). Additionally, epigenetic reprogramming initiated by *Arid1a* deletion improves mammalian regeneration in multiple tissues and thus may be a potential target to promote tissue repair after injury (Sun et al., 2016). Although recent advances have been made in *Arid1a* related to embryonic development, tissue repair, cell aging, apoptosis, and tumor formation, it remains unclear whether manipulating *Arid1a* is sufficient to enable axon regeneration or increase RGC survival following optic nerve injury *in vivo*.

In this study, we first examined how the expression level of *Arid1a* in sensory neurons and RGCs changed in response to axotomy *in vivo*. Functionally, we assessed whether conditional knocking out *Arid1a* in adult RGCs could promote neuronal survival or axon regeneration *in vivo*. Finally, we performed joint profiling of chromatin accessibility and gene expression in mature RGCs to gain mechanistic insights underlying *Arid1a* for modulating neuronal survival after optic nerve injury.

## Materials and Methods

### Mice

All mouse experiments were approved by the Animal Committee of the Institute of Zoology, Chinese Academy of Sciences, Beijing, China. Unless otherwise noted, male 6–8-week-old CD-1 IGS mice were used in experiments involving dorsal root ganglion (DRG) and sciatic nerve, and male 6–8-week-old C57BL/6 mice were used in experiments involving the retina and optic nerve. The *Arid1a^f/f^* mouse strain (JAX stock#027717) was a kind gift from Dr. Zhong Wang’s laboratory at the University of Michigan. All *Arid1a^f/f^* mice used in our experiments were 6–8 weeks male or female, and genotyped by PCR using primers (sequence: forward: TGGGCAGGAAAGAGTAATGG; reverse: AACACCACTTTCCCATAGGC) provided by The Jackson Laboratory. All animal anesthetized *via* intraperitoneal injection of ketamine (100 mg/kg) and xylazine (10 mg/kg) diluted in sterile saline. Details of the surgeries are described below.

### Sciatic Nerve Injury

Under deep anesthesia with a mixture of ketamine and xylazine, bilateral sciatic nerves were exposed and transected with spring scissors right below the pelvis. The mouse was euthanized, and bilateral L4/5 DRGs were collected at the expected time point after the surgery.

### RNA Extraction and qRT-PCR

Total RNA was extracted using Trizol reagent (Invitrogen). After extraction, 1 μg of total RNA was transcribed into cDNA using oligo (dT) primers (TransScript One-Step gDNA Removal and cDNA synthesis kit, TRANS) according to the manufacturer’s protocol. For real-time PCR analysis, using an SYBR^®^Premix Ex Taq™ (TliRNaseH Plus, Takara), 25 ng of cDNA, and 0.5 mM primers in a final volume of 20 μl. According to instructions, the PCR steps were performed 30 s initial pre-denaturation at 95°C, followed by 45 cycles of each 10 s at 94°C, 30 s at 60°C, 30 s at 72°C and samples were run in triplicate. The analysis of RT-PCR used the 2-ΔΔCT method. Primers for real-time RT-PCR are as follows:* Gapdh* forward: 5′-AGGTCGGTGTGAACGGATTTG-3′, *Gapdh* reverse: 5′-TGTAGACCATGTAGTTGAGGTCA-3′, *Arid1a* forward: 5′-TCCCAGCAAACTGCCTATTC-3′, *Arid1a* reverse: 5′-CATATCTTCTTGCCCTCCCTTAC-3′.

### AAV2 Virus Injection and Optic Nerve Crush

Intravitreal injections of AAV2 viruses, ONC, and RGC axon labeling were performed as previously described (Park et al., 2008). Animals were first anesthetized, 1.5 μl of AAV2 virus or Alexa Fluor 555-conjugated CTB (1 μg/μl, Thermo Fisher Scientific, Waltham, MA, USA) was then injected into their right vitreous humor with a Nanoliter syringe. CTB-555 injection was performed 2 days before the mouse was sacrificed to trace regenerating RGC axons. The optic nerve was exposed intraorbitally and crushed with forceps (Dumont #5, Fine Science Tools) for 5 s approximately 1 mm behind the optic disc. The mice were transcardially perfused with 4% paraformaldehyde (PFA). Dissected right optic nerve and bilateral retinas were post-fixed in 4% PFA overnight. pCMV-GFP and pCMV-Cre were from Vigene Bioscience. The titers of AAV2-CMV-Cre and AAV2-CMV-GFP were 1.62 × 10^13^ vg/ml and 3.2 × 10^13^ vg/ml, respectively.

### Whole-Mount Optic Nerve Preparation

Dehydration and clearing of optic nerves were performed based on previous studies (Erturk et al., 2012; Luo et al., 2013). Briefly, optic nerves were dehydrated in tetrahydrofuran (THF, 50%, 70%, 80%, 100%, and 100%, v/v % in distilled water for 20 min each, Sigma–Aldrich, St. Louis, MO, USA), then the nerves were transferred in benzyl benzoate/benzyl alcohol (BBBA, 2:1 in volume, Sigma–Aldrich, St. Louis, MO, USA) clearing solution for 20 min.

### Immunostaining

Frozen DRG or retinal sections of 20 μm thick were obtained with a cryostat. Whole-mount retinas were radially cut into a petal shape. After being washed with PBS at room temperature for 15 min, sections or whole-mount retinas were immunostained overnight at 4°C with primary antibody: mouse anti-βIII tubulin (Tuj1; 1:500, Biolegend 801202), rabbit anti-ARID1A (1:500, Atlas HPA005456), rabbit anti-Tuj1 (1:500, Biolegend 845501), mouse anti-Cre (1:500, Millipore MAB3120). Then, tissues were incubated for 2 h at room temperature with Alexa Fluor conjugated secondary antibodies (1:500, Alexa 488, Alexa 594, Alexa 647, Invitrogen). All antibodies were diluted in PBS, containing 0.3% Triton X-100 and 2% BSA. Three to four times of 15 min wash with PBS containing 0.3% Triton X-100 was performed following each antibody incubation.

### Imaging

Different experiments (sections and whole-mount) were imaged using Zeiss 780 or Nikon A1 with a motorized stage. All the images were z-projected to maximal intensity for quantification.

### ARID1A Signal Analysis

To analyze the protein level of *Arid1a* in all neurons, retinal or DRG sections were stained with rabbit anti-ARID1A and mouse anti-Tuj1 antibodies following the steps mentioned above (see “Immunostaining” section). Fluorescence intensity was measured using ImageJ, and the background fluorescence intensity was subtracted. Only Tuj1^+^ cells were measured.

### Analysis of RGC Transduction Rate

For quantification of RGC transduction rate, uninjured right retinas were taken from *Arid1a^f/f^* mice 2 weeks after intravitreal AAV2-Cre injection, and stained with mouse anti-Cre and rabbit anti-Tuj1 antibodies following the steps mentioned above (see “Immunostaining” section). For each mouse, the RGC transduction rate was calculated by dividing the average number of Cre^+^/Tuj1^+^ cells in one field by the average number of Tuj1^+^ cells.

### Measurement of RGC Survival Rate

To quantify the RGC survival rate, injured and intact retinas were taken from *Arid1a^f/f^* mice after AAV2 virus injection and ONC, then were stained with rabbit anti-Tuj1 antibody. For each flat-mounted retina, at least 14 fields were analyzed randomly obtained from the peripheral regions. RGC survival rate was calculated by dividing the average number of Tuj1^+^ cells in one field in the injured retina (right) by that in the uninjured retina (left). Only cells in the ganglion cell layer were counted.

### RGC Purification

The dissected retinas were dissociated in EBSS containing papain and DNase (Worthington) for 20 min at 37°C, with gentle shaking every 5 min, then digestion was stopped by adding Ovomucoid/EBSS (Worthington). The dissociated retinal cells were incubated with the FC block antibody (1:100, BD Biosciences 553141), and then stained with Thy1.2-PE antibody (1:100, Invitrogen 12-0902-82) and DAPI. Fluorescence-activated cell sorting (FACS) sorting was performed with a BD FACSAria IIIu instrument.

### RNA-Sequencing and ATAC-Sequencing

RNA-sequencing (RNA-seq) libraries from the purified RGCs were prepared using the Illumina RNA-Seq Preparation Kit. Total RNA sample QC was measured by Agilent 2100 Bioanalyzer (Agilent RNA 6000 Nano Kit). ATAC-sequencing (ATAC-seq) libraries were prepared from the purified RGCs using TruePrep DNA Library Prep Kit V2 for Illumin(Vazyme, TD-502). Libraries were sequenced on HiSeq X Ten, and 150 bp paired-end reads were generated. Raw read data were firstly filtered by using Trimmomatic (v. 0.36), and quality-controlled by using FastQC (v. 0.11.7; Andrews, 2010; Bolger et al., 2014). High-quality reads of RNA-seq were quantified using Salmon (v.1.0.0) with the parameter “—validate mappings —gcBias”, and the gene expression matrix was generated by tximport (v1.14.0; Soneson et al., 2015; Patro et al., 2017). Differential gene expression analysis was conducted using DESeq2 (v1.26.0) and increased and decreased expression were defined by log_2_(fold-change) > 0 and *P*-adjust < 0.05, and log_2_(fold-change) < 0 and *P*-adjust < 0.05, respectively (Love et al., 2014). High-quality reads of ATAC-seq were aligned using Bowtie 2 (v2.3.5.1) with the parameters “-X 2000—local” (Langmead and Salzberg, 2012). Diffbind (v.2.14.0) was then used for quantitative comparison of ATAC-seq data: peaks with increased and decreased chromatin accessibility after *Arid1a* deletion was defined by Fold > 0 and FDR < 0.05, or Fold < 0 and FDR < 0.05, respectively (Stark and Brown, 2011). Peak annotation was performed using ChIPseeker (v.1.22.1) at the genome level, and the promoter region was defined as ± 1,000 bp from TSS (Yu et al., 2015). Gene enrichment analysis was performed using clusterProfiler (v3.14.2; Yu et al., 2012). The mouse reference genome sequence (vM23) and gene annotation (vM23) were downloaded from GENCODE^1^. For genome browser representation, data in bigwig files (RPGC normalization) generated by deepTools (v3.3.1) were visualized using IGV (v. 2.4.10; Ramírez et al., 2014). DeepTools “computeMatrix,” “plotHeatmap,” and “plotProfile” functions were used to generate heatmaps and profile plots. For HOMER (v4.11) *de novo* motif analysis: findMotifsGenome.pl (-size 200 -S 10) was used for motif finding (Heinz et al., 2010). RGT-HINT was employed to execute differential footprints analyze (Li et al., 2019). RPKM values of ATAC-seq analysis were derived as follows: [(read-counts)/(region-length in kb)]/(total mapped reads in Mb). STRING (v11.0) and Cytoscape (v3.7.2) were adopted to create protein-protein interactions and visualization (Shannon et al., 2003). The transcription factors (TFs) regulating *Socs3* expression were explored with TRRUST (v2; Han et al., 2018).

### Statistical Analysis

For Statistics, we used GraphPad Prism software and data analyzed using a two-tailed unpaired student’s *t*-test to compare the differences. Data were shown as means ± Standard Error of Mean (SEM) of the mean. Statistical significance was set as *p* < 0.05.

## Results

### Axotomy Induces Robust Decreases of *Arid1a* in Sensory Neurons and Retinal Ganglion Cells

The peripheral nervous system (PNS) neurons spontaneously regenerate their axons and possess a strong growth capacity after injury, compared to CNS neurons (Chandran et al., 2016). To determine the role of *Arid1a* in the PNS axon injury response, we first investigated whether the expression levels of *Arid1a* in DRG neurons change day 1 or day 3 after sciatic nerve transection in CD-1 IGS mice. The results from quantitative real-time PCR (qRT-PCR) analysis showed mRNA levels of *Arid1a* dramatically decreased after SNI compared to the control group (Supplementary Figure S1A). Furthermore, double immunostaining of DRG sections with antibodies against ARID1A and Tuj1 confirmed that the protein levels of *Arid1a* gradually decreased in both cytoplasm and nucleus of DRGs after SNI (Supplementary Figures S1B–D), suggesting that both ARID1A protein synthesis and ARID1A protein transportation to the nucleus were reduced in DRG neurons after axonal injury.

**Figure 1:**
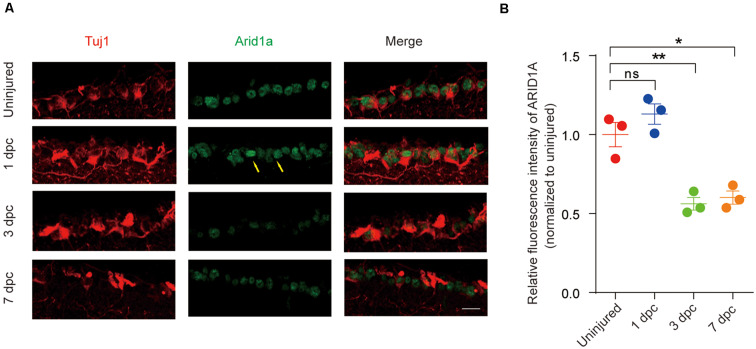
*Arid1a* expression is downregulated in retinal ganglion cells (RGCs) after optic nerve crush (ONC). **(A)** Retinal sections from C57BL/6 mice of different time points post-crush were collected and stained with Tuj1 (red), and ARID1A (green). Scale bar: 20 μm. **(B)** Quantification of relative fluorescence intensity of ARID1A staining in RGCs. Uninjured was used for normalization, for 1 dpc, ns, no significance; for 3 dpc, **p < 0.01; for 7 dpc, *p < 0.05. RGCs were analyzed from at least eight non-adjacent retinal sections for each animal, from three mice per group.

To further investigate *Arid1a* is involved in nerve injury, we used an established ONC model to examine the pattern of *Arid1a* in response to CNS axon injury. Immunostaining analysis of retinal sections showed that ARID1A was observed in RGCs. The protein levels of *Arid1a* were unchanged at day 1 after ONC but drastically decreased at day 3 and day 7 after ONC in C57BL/6 mice (Figures 1A,B). Similar results were obtained through detecting *Arid1a* transcripts in purified RGCs at different time points after ONC with RNA-seq (data not shown). These results clearly show that axon injury induces a dramatic decrease of *Arid1a* in axotomized adult neurons, which suggests that *Arid1a* may play certain roles in regulating axon regeneration or neuronal survival after nerve injury.

### Loss of *Arid1a* Promotes RGC Survival But Not Axon Regeneration *in vivo*

**Figure 2:**
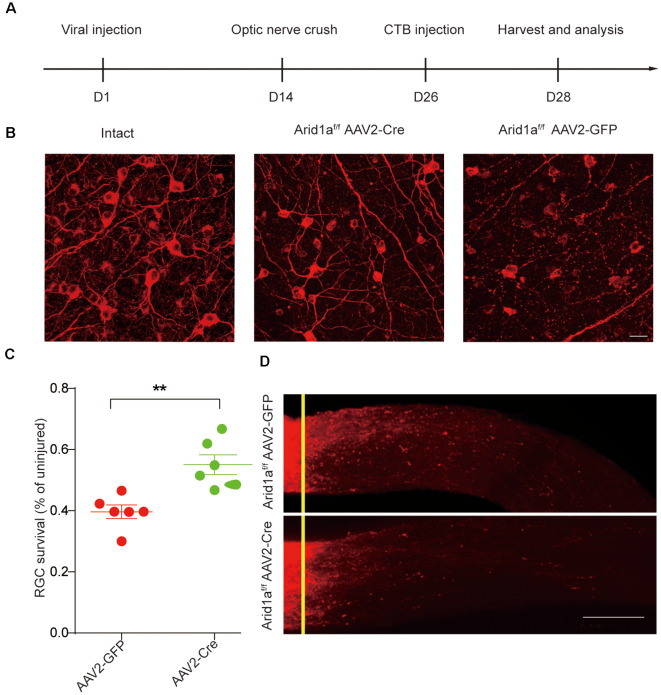
Loss of *Arid1a* promotes RGC survival. **(A)** Timeline of the experimental procedures. **(B)** Representative images of flat whole-mount retina stained with anti-Tuj1 from uninjured (intact), injured control (AAV2-GFP, Arid1a-WT), and injured *Arid1a^f/f^* mice (AAV2-Cre, Arid1a-cKO). Scale bar: 20 μm. **(C)** Quantification of RGC survival at 14 days post-crush, expressed as a percentage of the average number of Tuj1+ RGCs in the contralateral (intact) retina. **p < 0.01. *n* = 6 mice in each group. **(D)** Representative confocal images of CTB-labeled optic from *Arid1a^f/f^* mice with intravitreal injection of AAV2-GFP or AAV2-Cre viruses. *n* = 6 mice in each group. The yellow lines indicate the nerve crush sites. Scale bar: 100 μm.

To further characterize the functional roles of *Arid1a* in axon regeneration and RGC survival, we performed *in vivo* conditional *Arid1a* knockout (*Arid1a* cKO) in mature RGCs by intravitreal injection of adeno-associated virus serotype 2 expressing Cre (AAV2-Cre) in *Arid1a^f/f^* mice, while AAV2-GFP was used as a control. Two weeks after the injection, immunostaining of whole-mount retinas with antibodies against Tuj1 and Cre showed that the virus transduction rate in RGCs was about 89% (Supplementary Figure S2A). Immunostaining analyses of *Arid1a* in retina cross-sections further showed that its protein levels in Tuj1 positive cells significantly decreased 2 weeks after AAV2-Cre injection, indicating *Arid1a* deletion in RGCs (Supplementary Figures S2B,C). Next, we analyzed the roles of *Arid1a* on axon regeneration and neuroprotection. The viruses injected mice were subjected to a standard ONC procedure 2 weeks after the injection, followed by a cholera toxin B subunit (CTB) injection to analyze neuronal survival and axon regeneration (Figure 2A). Significantly, we observed that 55 ± 5% of RGCs survived in *Arid1a* cKO mice, whereas only 35 ± 5% in control mice injected with AAV2-GFP (Figures 2B,C). However, in both control and *Arid1a* cKO mice, almost no CTB-labeled axon was observed beyond the crush site (Figure 2D). These results together verify that *Arid1a* deletion selectively affects RGC survival, but not axon regeneration.

### Transcriptome Analysis Reveals Dynamic Gene Expression Induced by Knocking Out *Arid1a* in Adult RGCs

To gain mechanistic insights into the effects of *Arid1a*, we employed a combinatorial assay that jointly profiles transcriptome and chromatin accessibility. To this end, we injected AAV2-Cre (hereafter called cKO) or AAV2-GFP (hereafter called WT) into the vitreous body of *Arid1a^f/f^* mice, waited 2 weeks, and performed ONC. Three days later, axotomized retinas were dissected out, and dissociated cells were subjected to FACS for RGC enrichment (Supplementary Figure S3A). FACS gating parameters were optimized to select cells based on size, viability (DAPI-negative cells), and Thy1.2-PE+ (a specific marker of RGCs) signal (Supplementary Figure S3B). Almost all FACS-purified cells were Tuj1 positive by immunostaining (Supplementary Figure S3C).

**Figure 3:**
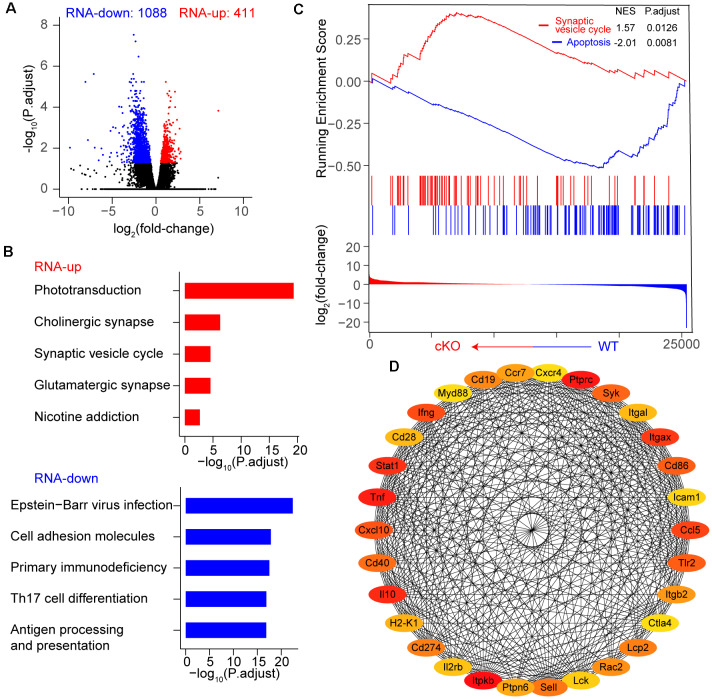
Transcriptome dynamic changes of RGCs after *Arid1a* deletion. **(A)** Volcano plot of log_2_ (fold-change) vs. –log_10_ (P.adjust) representing gene expression changes between WT and *Arid1a* cKO RGCs. **(B)** Kyoto encyclopedia of genes and genomes (KEGG) analysis of these differentially expressed genes in **(A)**, the terms with the five highest –log_10_ (P.adjust) are shown as a bar plot. **(C)** Gene set enrichment analysis (GSEA)-KEGG pathway analysis of RNA-seq data. **(D)** The protein-protein interactions of differentially expressed genes, only the 30 genes with the highest degree (low values to dark colors) are shown in a circular layout.

We first performed RNA sequencing (RNA-seq) to characterize the cellular state of adult RGCs upon *Arid1a* cKO. RNA-seq analysis showed that *Arid1a* deletion in adult RGCs caused dramatic changes in gene expression (Figure 3A). A total of 1,499 differentially expressed genes (DEGs, *P*-adjust < 0.05), including 411 upregulated and 1,088 downregulated genes, were identified in *Arid1a* cKO RGCs compared to WT RGCs (Figure 3A and Supplementary Table S1). To identify the biological pathways perturbed by *Arid1a* deletion, we subjected the DEGs to the Kyoto encyclopedia of genes and genomes (KEGG) analysis. The results showed that upregulated genes were involved in phototransduction, cholinergic synapse, and synaptic vesicle cycle, suggesting that *Arid1a* might associate with synapse transmission (Figure 3B). The characteristics of downregulated genes in *Arid1a* cKO RGCs were related to viral infection, cell adhesion, and immune response, etc. (Figure 3B). Furthermore, gene set enrichment analysis (GSEA) indicated that genes related to apoptosis were downregulated in *Arid1a* cKO RGCs compared to WT RGCs, whereas upregulated genes participated in the synaptic vesicle cycle (Figure 3C). To further explore mechanisms underlying RGC survival mediated by *Arid1a* deletion, we performed the protein-protein interactions (PPIs) of differentially expressed genes. Strikingly, we observed some of the 30 genes with the highest degree shown in the circular layout were related to neuronal survival, such as *Stat1*, *Tlr2* (Figure 3D; Stivers et al., 2017; Liu et al., 2019).

### ATAC-Seq Analysis Shows Widespread Chromatin Accessibility Changes in *Arid1a*-Deleted RGCs

**Figure 4:**
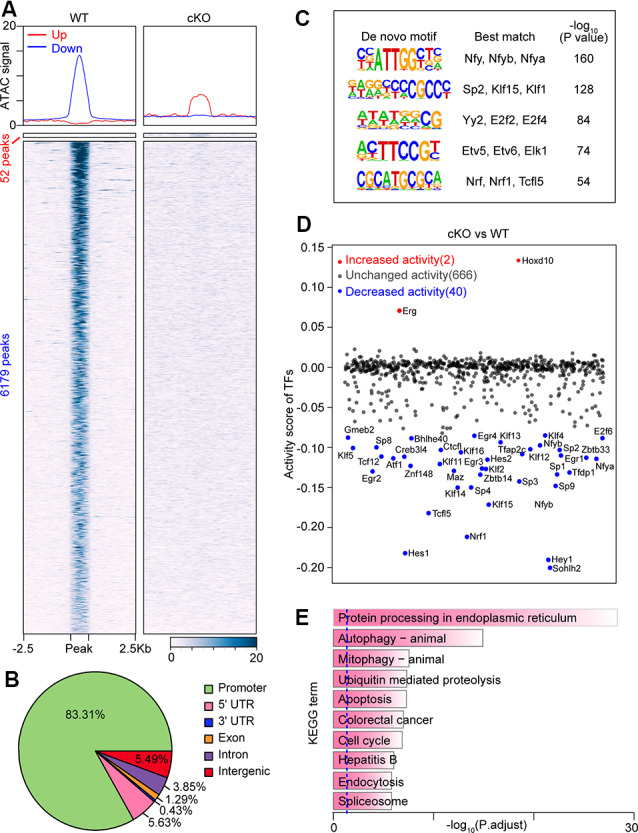
Loss of *Arid1a* leads to disturbed chromatin accessibility and binding of transcription factors (TFs) in RGCs. **(A)** Average profiles and heatmaps of chromatin accessibility within ±2.5 kb of groups of peaks. Up and Down: peaks with increased and decreased chromatin accessibility after *Arid1a* deletion in RGCs separately. **(B)** Pie chart showing the distribution of peaks with reduced ATAC signal caused by *Arid1a* loss at annotated genomic regions. **(C)** Sequence logos corresponding to enriched elements identified by *de novo* motif analysis of peaks with a decrease in chromatin accessibility at the promoter regions after *Arid1a* deletion in RGCs. **(D)** Scatter plot showing changes of TFs’ activity predicated using ATAC-seq data after *Arid1a* deletion in RGCs. **(E)** KEGG pathway enrichment analysis of genes annotated in these peaks defined in **(C)**.

*Arid1a*, as a core subunit of the SWI/SNF chromatin-remodeling complex, functions to interact with DNA and is directly involved in regulating chromatin plasticity (Sun et al., 2016). Next, to investigate how chromatin accessibility changes upon *Arid1a* deletion, we performed an assay for transposase-accessible chromatin using sequencing (ATAC-seq) of biological replicates (*n* = 2 for each group) of purified RGCs with and without *Arid1a* after ONC. We found that there were very similar chromatin-accessibility profiles for the biological replicates in each group (Supplementary Figures S4A,B). In contrast, the accessible chromatin landscapes in *Arid1a* cKO RGCs were significantly different from that of WT RGCs (Figure 4A; Supplementary Figure S4C). Furthermore, by quantifying signal intensities, 52 gained-open, and 6,179 gained-closed peaks were identified in *Arid1a* cKO RGCs compared with WT RGCs (Figure 4A). Moreover, genome annotation of gained-closed sites in *Arid1a* cKO RGCs exhibited a wide genomic distribution, including promoter regions, intergenic regions, introns, and exons. We also observed that 83.31% of these gained-closed peaks were mapped to promoter regions, which controlled the expression of associated genes (Figure 4B, Supplementary Figures S4D,E). We next used *de novo* motif analysis to identify the top five binding site motifs for TFs in ATAC-seq gained-closed peaks at promoter regions in *Arid1a* cKO RGCs (Figure 4C). Correspondingly, *Arid1a* deletion in adult RGCs led to a dramatic decrease in the activity of TFs (Figure 4D). KEGG analysis of these genes associated with gained-closed peaks at the promoter regions in *Arid1a* cKO RGCs revealed enrichment for terms related to autophagy and apoptosis (Figure 4E). For example, the previous study reported that the nuclear transcription factor E-26-like protein 1 (*Elk1*) overexpression decreased viability in primary neurons, whereas *Elk1* knockdown increased viability (Barrett et al., 2006). Together, these results indicate that *Arid1a* deletion in adult RGCs leads to significantly reduced transcriptional activation and chromatin accessibility, which are associated with RGC survival.

### SOCS3 Acts as a Downstream Target of ARID1A to Modulate Neuronal Survival

**Figure 5:**
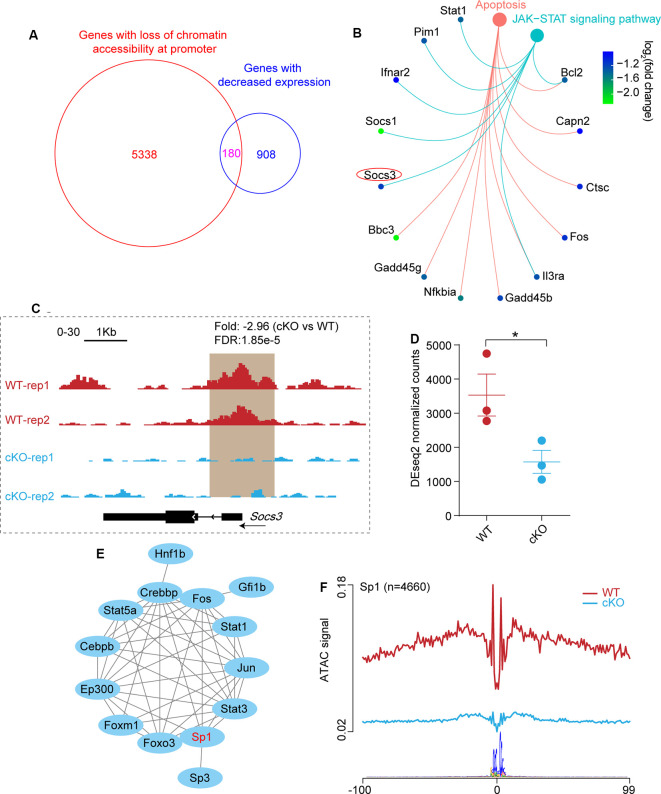
The absence of *Arid1a* protects RGCs from apoptosis through regulating the expression of *Socs3*. **(A)** Venn diagram shows the overlap of genes with significantly reduced ATAC-seq signal at the promoter regions and genes with decreased expression in *Arid1a* deleted RGCs. **(B)** KEGG analysis of these overlapped genes in (A), two terms related to apoptosis (P.adjust < 0.05) are shown. **(C)** Genome-browser view at the *Socs3* gene of different ATAC-seq data sets. Regions with a reduced ATAC-seq signal was highlighted in a brown background. **(D)** Statistical comparison of *Socs3* expression before and after *Arid1a* deletion in injured RGCs. *P < 0.05. **(E)** The protein-protein interactions of different TFs which regulate *Socs3* expression identifying with TRRUST. **(F)** Average profile showing differential footprinting of Sp1 at 4,660 sites.

By comparing the two sets of data, we observed a substantial overlap of genes associated with closed chromatin regions at the promoter (Supplementary Table S2) and downregulated genes (Supplementary Table S1) in *Arid1a* cKO RGCs (Figure 5A). Next, KEGG pathway analysis was performed to reveal characteristics of 180 overlapped genes (*P*-adjust < 0.05), and showed enrichment of pathways related to apoptosis (*Capn2*, *Ctsc*, and *Fos*, etc.) and JAK-STAT signaling pathway (*Socs1*, *Socs3*, and *Stat1*, etc.; Figure 5B). These findings support the idea that apoptosis programs may be repressed by *Arid1a* deletion, thereby leading to improved RGC survival.

Previous studies showed that *Socs3* deletion promoted RGC survival and axon regeneration (Smith et al., 2009; Luo and Park, 2012). Here, we observed significantly reduced chromatin accessibility at the *Socs3* locus in *Arid1a* cKO RGCs, indicating correlated with decreased gene expression (Figure 5C). Besides, quantitative transcriptome analysis by RNA-seq showed that there was a markedly reduced signal for *Socs3* in* Arid1a* cKO RGCs compared to WT RGCs (Figure 5D). By using the TRRUST tool, the PPI network analysis revealed that 14 TFs could regulate *Socs3* expression (Figure 5E), in which Sp1 had low transcriptional activity in *Arid1a* cKO RGCs (Figure 4D). Furthermore, the average profile showed that the ATAC signal of Sp1 was dramatically reduced in *Arid1a* cKO RGCs (Figure 5F). Notably, Sp1 has been shown to regulate *Socs3* expression and neuronal apoptosis (Wiejak et al., 2012; Qi et al., 2014). Together, these results suggest that *Socs3* may be a functional downstream target of *Arid1a*.

## Discussion

RGCs are the only output neuron of the retina, and their axons exit the eye to form the optic nerve, which relays visual information to retinorecipient areas in the brain (Laha et al., 2017). Like most CNS neurons in mature mammals, RGCs fail to regenerate in disease and after injury, eventually even leading to irreversible blindness (Benowitz et al., 2017; Laha et al., 2017). Up to date, there is indeed no effective therapy to restore visual functions after RGC axon injury. Over the last decade or two, much effort has been devoted to exploring visual repair strategies, including removing extracellular inhibitory factors [e.g., Nogo and its receptors (Fischer et al., 2004; Su et al., 2008)], and enhancing intrinsic regenerative capacity [e.g. *Pten*, *Klf4*, *Socs3*, and *Lin28* (Park et al., 2008; Moore et al., 2009; Smith et al., 2009; Wang et al., 2018)]. Generally, axon regeneration and RGC survival may involve distinct mechanisms (Goldberg et al., 2002). For instance, manipulation of some genes, such as *Pten* or *c-myc* (Park et al., 2008; Belin et al., 2015), in RGCs significantly promotes neuronal survival and axon regeneration, and that of other genes, including *Klf4*, *Lin28* or *Gal-3* (Moore et al., 2009; Abreu et al., 2017; Wang et al., 2018), contributes to only one. Despite significant advances, the level of RGC survival and the extent of axon regeneration is still limited.

A previous study reported that epigenetic reprogramming mediated by *Arid1a* deletion improves mammalian regeneration and suggests strategies to promote tissue repair after injury (Sun et al., 2016). We, therefore, reasoned that *Arid1a* deletion in mature RGCs could also sustain neuronal survival and encourage axon regeneration after optic nerve injury. Our study first revealed that the mRNA (data not shown) and protein levels of *Arid1a* in a mixed population of RGCs markedly decreased at day 3 and day 7 after optic nerve injury. However, it is worth noting that following optic nerve injury, no significant changes of *Arid1a* mRNA were observed in some subtypes of RGCs (Bray et al., 2019; Tran et al., 2019). Generally, RGCs are a heterogeneous population of cells divided into different subtypes based on functional, morphological, and molecular features (Sanes and Masland, 2015). We thus consider that this discrepancy may be caused by the different RGC populations of the samples. We next showed that conditionally knocking out *Arid1a* results in a markedly increased survival rate of RGCs 2 weeks after ONC. In contrast, *Arid1a* deletion was insufficient to boost neurite outgrowth and axon regeneration after optic nerve injury. These findings indicate that the absence of *Arid1a* has a potent neuroprotective effect on RGC apoptosis induced by axon injury, but insignificantly affects axon growth. A more recent study showed that few RGCs die during the first 3 days after optic nerve injury, ~70% die over the next 5 days, and nearly all are lost within a month (Tran et al., 2019). Therefore, at the early stage of axon injury, there may be a fairly narrow therapeutic window in which RGCs can be stimulated to regenerate axons while still relatively healthy. It is possible to extend the window for treatments that might stimulate axon regeneration *via* sustaining RGC survival. For example, *Bax* deletion leads to up to 80% of RGCs to survive 8 weeks after optic nerve injury and enables RGCs to regenerate axons even despite postponed initiation of CNTF-induced regenerative program for several weeks (Yungher et al., 2017). Besides, a few studies suggest that partial visual functional recovery might be attributed to only a small percentage of RGCs to regenerate axons back to the original targets despite full-length axon regeneration (de Lima et al., 2012; Lim et al., 2016). Thus, strategies increasing neuronal survival *via* distinct underlying mechanisms are still necessary to improve combinatorial therapies for visual recovery after optic nerve injury. Our study provided clear and strong evidence that deleting *Arid1a* can promote neuronal survival, suggesting a new protective strategy for RGC loss associated with axon injury.

As key roles in axon growth of RGCs during development, transcription factors might be manipulated to enhance RGC survival and optic nerve regeneration in adults. Recent studies reported that overexpression of *c-Myc* or knockdown of *Klf9* significantly promotes neuronal survival and axon regeneration after ONC (Belin et al., 2015; Apara et al., 2017). Additionally, *Klf4* deletion or *Lin28* overexpression produces optic nerve regeneration but not RGC survival (Moore et al., 2009; Wang et al., 2018). Tet signaling is also required for induced axon regeneration in adult RGCs with the specific involvement of *Tet1* (Weng et al., 2017). Interestingly, *Sox11* overexpression in RGCs boosts robust axon regeneration in a subset of RGCs after optic nerve injury but also kills α-RGC (Norsworthy et al., 2017). A recent study showed that deleting *Arid1a*, a core subunit of* the* SWI/SNF chromatin-remodeling complex substantially improves mammalian regeneration and soft tissue healing (Sun et al., 2016). In this study, we found that the deletion of *Arid1a* promotes RGC survival after optic nerve injury by regulating chromatin accessibility. Together, our work adds another important landmark toward intrinsic epigenetic reprogramming mechanisms that increase RGC survival following optic nerve injury in adults.

Further, we performed a co-assay of ATAC-seq and RNA-seq to explore intrinsic molecular mechanisms by which deleting *Arid1a* can promote RGC survival* in vivo* after optic nerve injury. We observed that *Arid1a* deletion leads to significantly reduced chromatin accessibility and transcriptional activation in adult RGCs. Importantly, joint profiling showed that *Arid1a* deletion significantly reduces ATAC-seq signal at *Socs3* loci, and decreased RNA-seq signal of *Socs3* mRNA. Besides, *Arid1a* deletion in adult RGCs led to a reduced transcriptional activity of Sp1 required for *Socs3* activation. These results suggested that *Socs3* may be a functional downstream target of *Arid1a*. The deletion of *Socs3* has been shown to promote dramatic axon regeneration and massive RGC survival following optic nerve injury in a *gp130*-dependent manner (Smith et al., 2009; Luo and Park, 2012). Mechanistically, SOCS3 acts as a negative feedback signal by inhibiting JAK and STAT3 activation and phosphorylation, limiting the response to cytokine and growth factors signaling. Thus, the injection of CNTF in *Socs3* deletion mice further increases the extent of axon regeneration (Smith et al., 2009). Interestingly, while *gp130* deletion abolishes the axon regeneration-promoting effect of *Socs3* deletion, a partial neuronal survival effect is preserved in *Socs3* and *gp130* double deletion (Smith et al., 2009). In our study, *Arid1a* deletion promotes neuronal survival but not axon regeneration, which may be partially mediated by JAK/STAT3/SOCS3 pathway. Besides, KEGG analysis showed that apoptosis programs might be repressed by *Arid1a* deletion, leading to increased RGC survival. However, we also observed that some KEGG terms were related to viral infection and immunity. Although the viral titers were enough for a high transduction rate in this work, there was still a slight difference between the two viruses. A previous study suggested that AAV could cause innate immune responses (Rogers et al., 2011). Also, animals were subjected to a complex operation procedure, including ONC and AAV injection, which might lead to immune response activation. Thus, we consider that these KEGG terms may be associated with different viral titers and operation procedures. For a better understanding of the functional role, it will be interesting to determine whether this or other mechanisms mediate the RGC survival effect of *Arid1a* deletion after injury.

In light of recent successes in AAV-mediated gene therapy in retinal diseases (Tan et al., 2009; Busskamp et al., 2010), gene therapy is recommended to be designed specifically for multiple therapeutic targets to achieve an optimal outcome for neural recovery. In summary, our results provide a new therapy for gene intervention to effectively decrease the loss of RGCs after optic nerve injury. Because, here we have only shown that deleting *Arid1a* promotes RGC survival 2 weeks after injury, to identify its clinical relevance, further works are needed to investigate whether the absence of *Arid1a* could save damaged RGCs after a long-term injury.

## Data Availability Statement

The original RNA-seq and ATAC-seq data from this study are available at the NCBI Gene Expression Omnibus (http://www.ncbi.nlm.nih.gov/geo/) under accession number GEO: GSE147844. All datasets generated for this study are included in the article/Supplementary Material.

## Ethics Statement

The animal study was reviewed and approved by The Animal Committee of the Institute of Zoology, Chinese Academy of Sciences, Beijing, China.

## Author Contributions

S-GY and C-ML: conception and design, collection and assembly of data, data analysis and interpretation, manuscript writing, and final approval of the manuscript. X-QP, S-KD, C-PL, P-PL, Z-MW, H-ZD, and Z-QT: collection and assembly of data.

## Conflict of Interest

The authors declare that the research was conducted in the absence of any commercial or financial relationships that could be construed as a potential conflict of interest.

## Footnotes

^1^https://www.gencodegenes.org/
