# Supplementary material for: Loss of Arid1a Promotes Neuronal Survival Following Optic Nerve Injury
Source: Front Cell Neurosci. 2020 May 15;14:131. doi: 10.3389/fncel.2020.00131 (PMC7326083; doi:10.3389/fncel.2020.00131)

**Loss of *Arid1a* Promotes Neuronal Survival Following Optic Nerve Injury**

Xue-Qi Peng 1,2,3†, Shang-Kun Dai 1,2,3†, Chang-Ping Li 1,2,3, Pei-Pei Liu 1,3,
Zhi-Meng Wang 1,2,3, Hong-Zhen Du 1,3, Zhao-Qian Teng 1,2,3, Shu-Guang Yang 1,4*
and Chang-Mei Liu 1,2,3*

1. State Key Laboratory of Stem Cell and Reproductive Biology, Institute of Zoology, Chinese Academy of Sciences, Beijing, China.

2. Savaid Medical School, University of Chinese Academy of Sciences, Beijing 100049, China.

3. Institute for Stem Cell and Regeneration, Chinese Academy of Sciences, Beijing 100101, China.

4. Department of Orthopedic Surgery, The Johns Hopkins University School of Medicine, Baltimore, MD 21287, USA.

*†*These authors contributed equally to this paper.

*****Correspondence author: S-G.Y. (lionamms@hotmail.com), C-M.L. ([liuchm@ioz.ac.cn](mailto:liuchm@ioz.ac.cn))

**Figure legend**

**Figure S1. Downregulation of ARID1A in DRG neurons after sciatic nerve injury**

1. *Arid1a* mRNA levels in L4/L5 DRG from CD-1 IGS mice without injury (uninjured), and,

1day, 3 days post sciatic nerve crush detected by q-PCR. Results are normalized to GAPDH, for 1 dpc, ns=no significance; for 3dpc, * p<0.05, n=3 mice in each group.

1. DRG sections from CD-1 IGS mice without injury (uninjured), and 1 day, 3 days after

sciatic nerve crush stained with Tuj1 (red), and ARID1A (green). Scale bar: 50 μm.

1. Quantification of relative fluorescence intensity of ARID1A staining in cytoplasm of

DRG neurons. Uninjured was used for normalization, for 1 dpc, * p<0.05; for 3 dpc, *** p<0.001, n=3 mice in each group, cell analyses were performed on 3-4 nonconsecutive DRG sections for each mouse.

1. Quantification of relative fluorescence intensity of ARID1A staining in nucleus of DRG

neurons. Uninjured was used for normalization, for 1 dpc, * p<0.05; for 3 dpc, *** p<0.001, n=3 mice in each group, cell analyses were performed on 3-4 nonconsecutive DRG sections for each mouse.

**Figure S2. ARID1A is deleted after AAV2-Cre virus intravitreal injection**

1. Representative confocal images of flat-mounted retinas *Arid1a^f/f^* mice with

intravitreal injection of AAV2-Cre stained with Tuj1 (green) and Cre (red). The bottom row images show enlarged images of the top row images. The infection rate of AAV2-Cre in RGCs was 89.85 % on average. n=2 mice. Scale bar, 1 mm in top images, and 50 μm in bottom images.

1. Retinal sections from *Arid1a^f/f^* mice with intravitreal injection of AAV2-GFP or AAV2-

Cre stained with Tuj1 (green), and ARID1A (red). Scale bar: 20 μm.

1. Quantification of relative fluorescence intensity of ARID1A staining in RGCs. AAV2-

GFP was used for normalization. **p<0.01. RGCs were analyzed from at least 13 non-adjacent retinal sections for each animal, from 3 mice per group.

**Figure S3. Isolation of** **Thy1.2-PE^+^ RGCs for RNA-sequencing and ATAC-sequencing**

1. Overview of RNA sequencing and ATAC sequencing from the purified RGCs.
2. Representative FACS plots illustrating the steps of RGCs purification. Dissociated

retinal cells were gated based on size and surface characteristics (forward scatter, FSC-A, x axis; side scatter, SSC-A, y axis; first graph from left). DAPI-positive neurons, staining dead cells depicted in blue, are excluded by sorting (second graph from left). Retinal cells without Thy1.2-PE were used as controls to set up the threshold for Thy1.2-PE+ cells (third graph). The last graph shows the population of Thy1.2-PE+ RGCs that were selected (4-5 retinas in each sample).

1. Representative confocal images of the purified RGCs stained with DAPI (gray) and

Tuj1 (green). Scale bar: 20 μm.

**Figure S4. *Arid1a* deletion reduces chromatin accessibility at promoters and gene expression.**

1. Correlation heatmap to evaluate the correlation between indicated samples.
2. Venn diagram shows the peaks’ overlap between indicated samples.
3. Average profiles and heatmaps of ATAC-seq data for RGCs plotted within ± 5Kb

from transcription start sites (TSS) of 60609 annotated genes and presented in RPGC (reads per genomic content).

1. Left graph, boxplot showing gene expression changes of different groups of genes:

transcript per million of genes (TPM > 0, 27584 genes) were divided into five bins, as indicated, from lowly expressed genes (Bin1) to highly expressed genes (Bin5); Right graph, boxplot showing the changes of promoter chromatin accessibility of different groups of genes defined previously.

1. GSEA analysis of association between changes of chromatin accessibility at indicated

regulatory elements and gene expression changes.

**Figure S1**


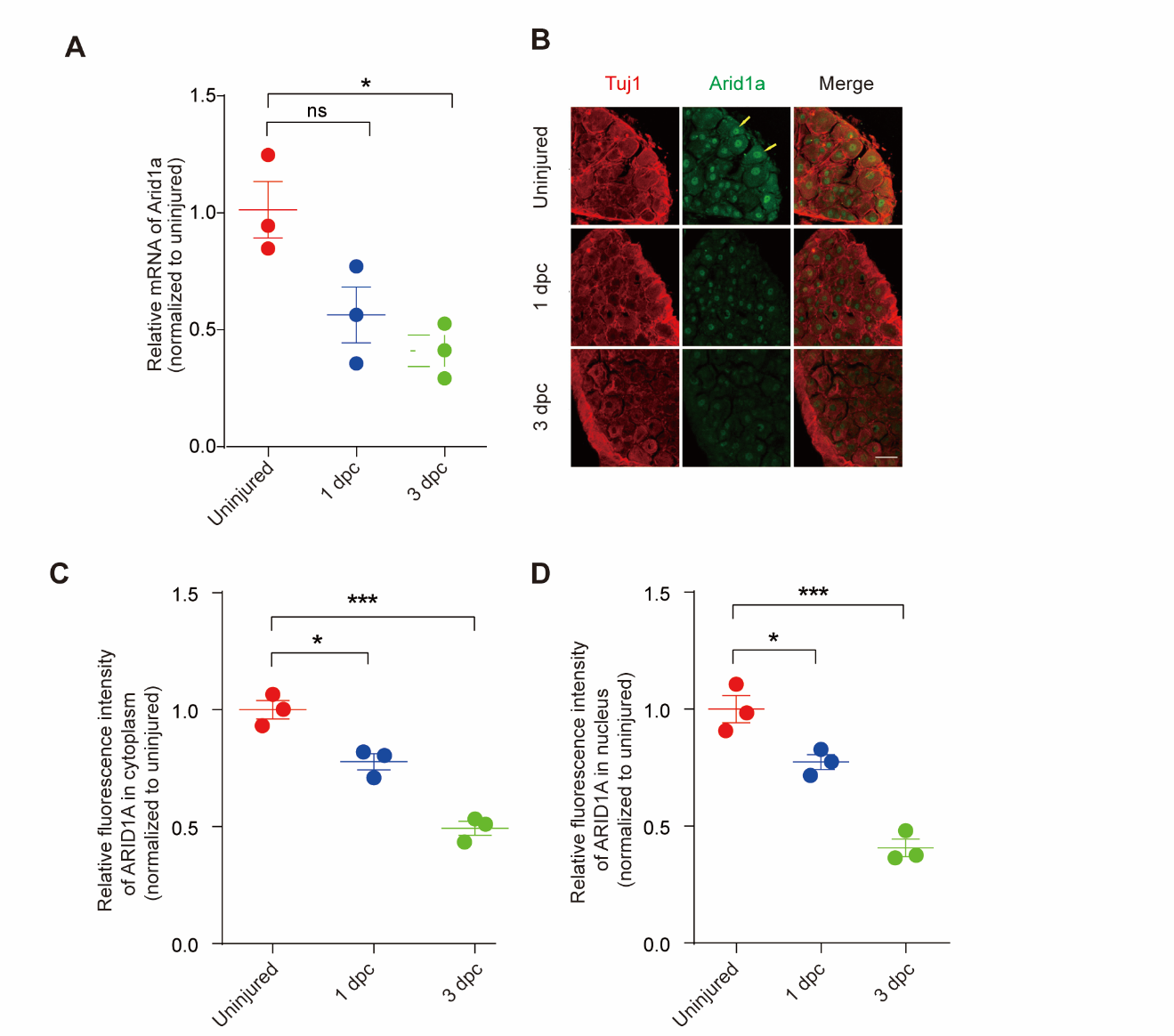


**Figure S2**


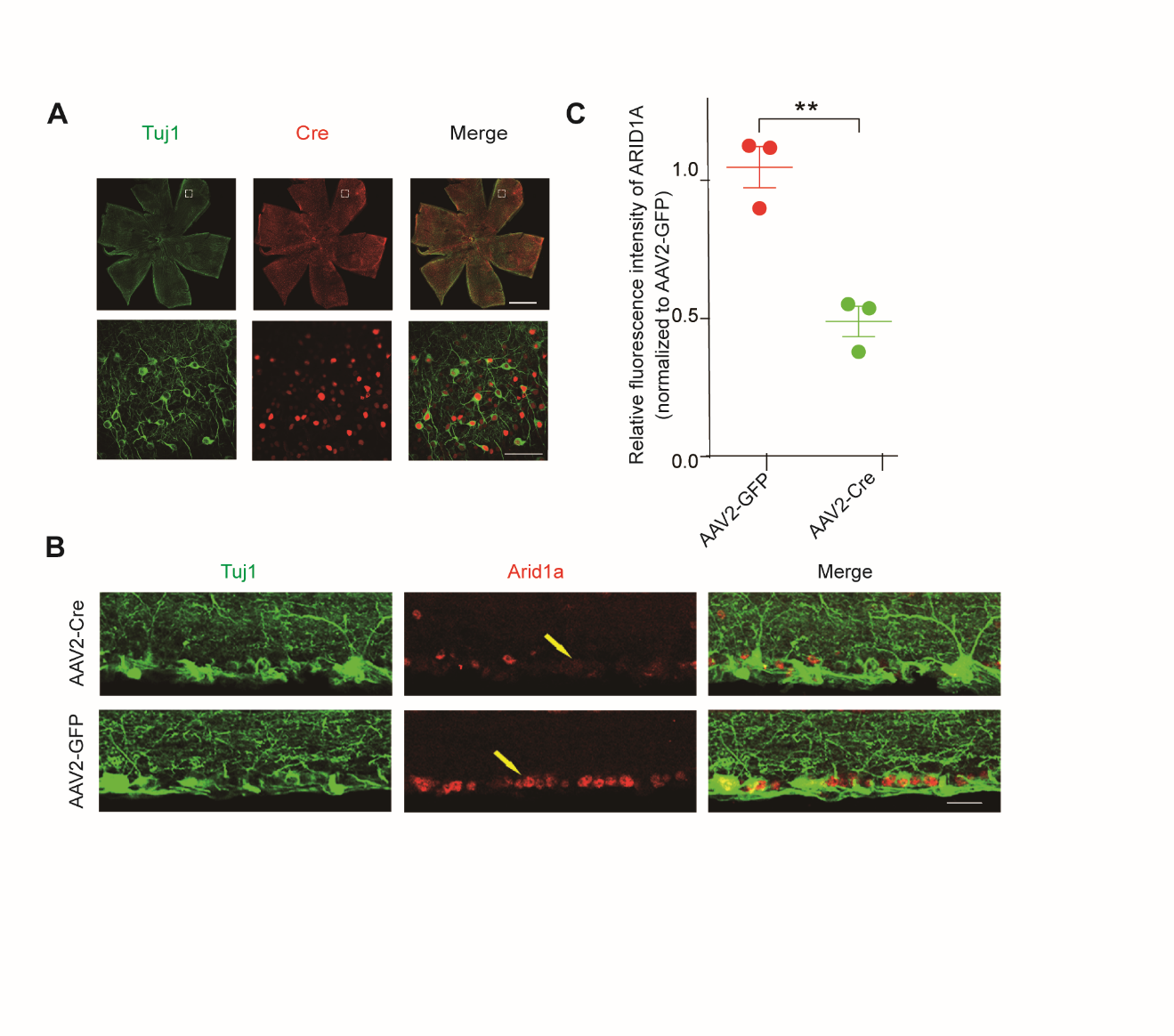


**Figure S3**

**
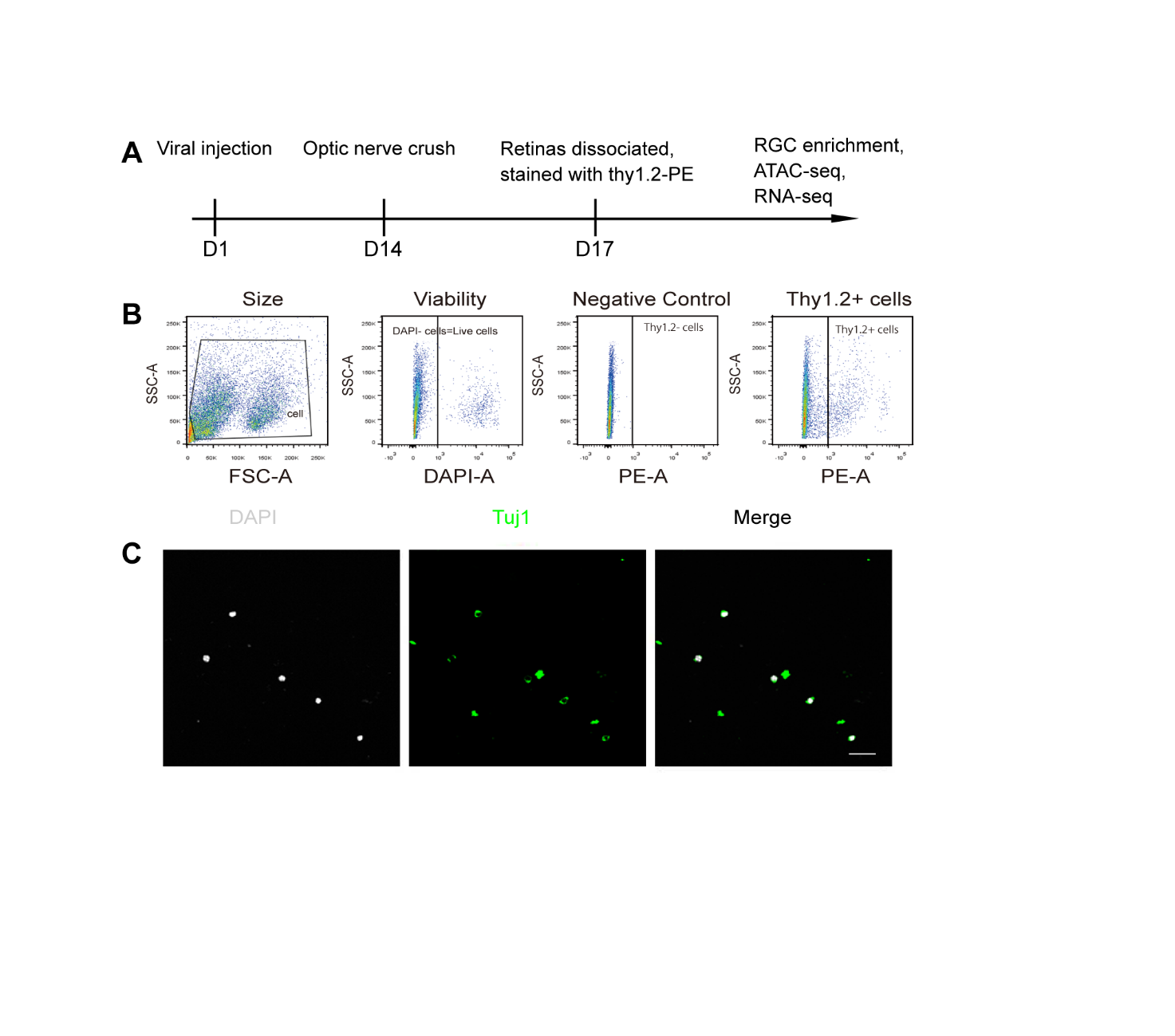
**

**Figure S4**


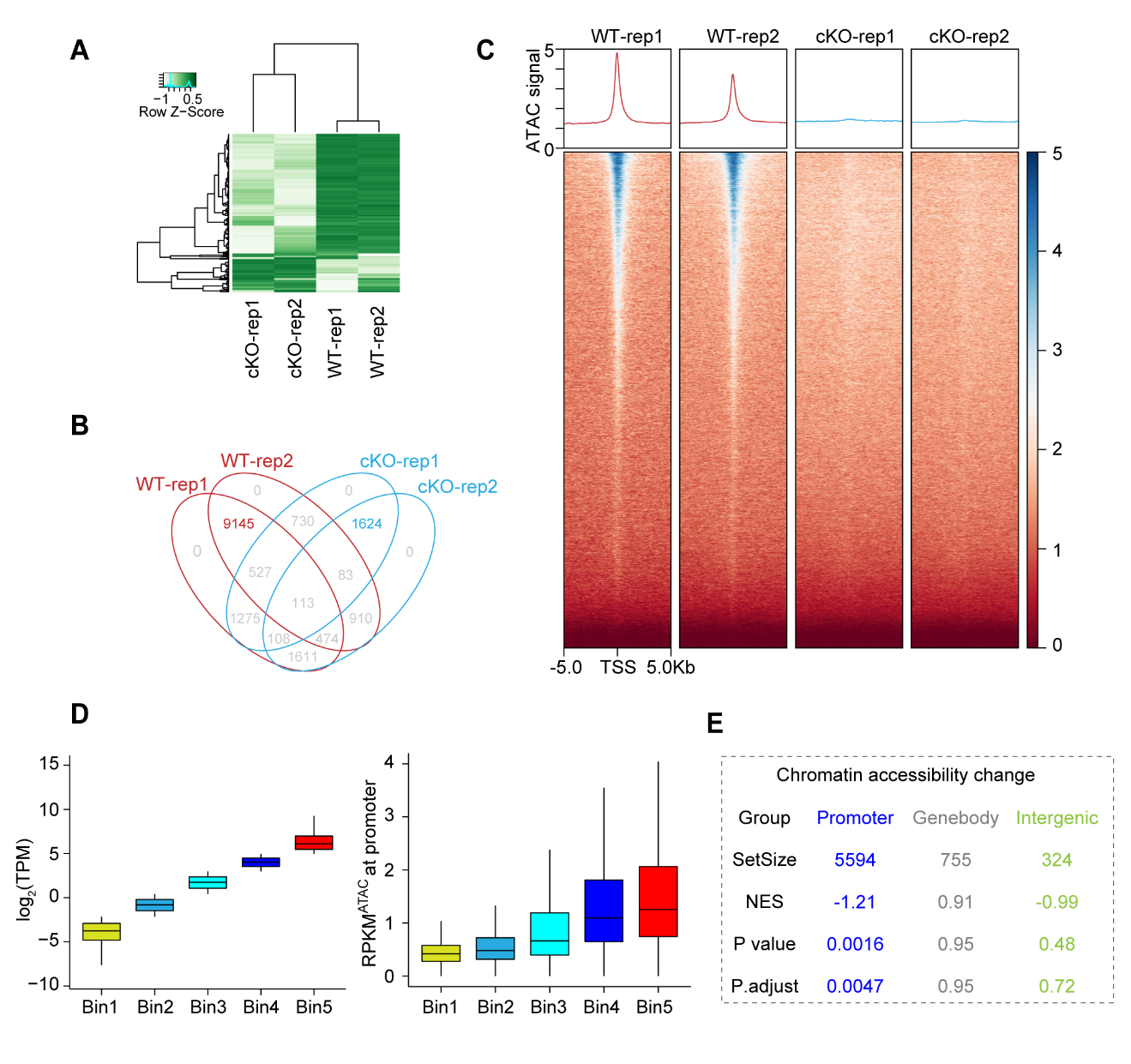

Supplement: Supplementary file 3 [file Data_Sheet_1.docx]
